# Progress in Diseases of the Nervous System

**Published:** 1901-11-23

**Authors:** 


### PROGRESS IN DISEASES OF THE NERVOUS SYSTEM.

**General Paralysis of the Insane.**—Important papers on the pathogenesis of general paralysis of the insane have been published by Robertson and Bruce. Robertson[1] says that nearly all authorities now recognise that antecedent syphilitic infection is the essential cause in a very large proportion of cases. The long interval of time which elapses between infection and the morbid process in the nervous system is a difficulty in admitting that there is a direct action of the syphilitic poison on the brain. Two theories have consequently arisen—the theory of the premature involution of the cortical cells, and that of a toxæmia. According to the former the syphilitic poison produces on the nerve cells of the cortex a certain impression in consequence of which these cells after some years become exhausted and undergo premature involution. Against this theory of premature involution Robertson urges the following—the clinical and pathological picture of general paralysis has no true resemblance to normal senile involution. Again, in general paralysis, the whole nervous system suffers, not merely certain functional groups of neurons, which it might be expected would be the case in premature involution. The theory, too, does not explain the unquestionably close association between general paralysis and tabes dorsalis, for the fact is now clearly established that the latter disease does not depend on degeneration of the cells of the posterior root ganglia. It does not accord with the phenomena of successive remission and recrudescence of the more active phase of the disease. On the other hand the evidence which has now been collected in support of the theory of a chronic toxæmia is exceedingly strong. Thus the vascular lesions which are not limited to any particular region of the nerve centres can only be produced by toxins circulating in the blood. The parts of the central nervous system that first give way are precisely those which exhibit the greatest degree of functional activity and have consequently received the largest amount of blood, and hence the greatest quantity of toxins. This chronic toxæmia is probably of gastro-intestinal origin, due to a partial breakdown of those forces by which the harmful development of the micro-organisms which constitute the ordinary flora of the alimentary tract is normally prevented. The part played by syphilis in the pathogenesis of general paralysis and tabes dorsalis is essentially that of altering the general immunity. Probably the only means by which it will be found possible to check the excessive growth of the gastro-intestinal bacteria is that of the employment of specific antitoxins. This theory of Robertson's is strongly supported by some observations of Bruce,[2] who first remarks that he has never yet seen a case of general paralysis in which one did not meet with intestinal and gastric disorders. Now, the characteristic temperature in general paralysis is a recurrent febrile attack every one or two weeks. Examination of the blood shows that to every rise of the thermometer there is a corresponding rise of leucocytes; in the first stage the hyperleucocytosis is often very high, sometimes 20,000 to 30,000 per cb.mm. Progressive cases of the disease in which there is no recurrent febrile temperature present every now and then a hyperleucocytosis, and these periods coincide with advances in the disease. In definite remissions of the disease, the leucocytosis falls to that of health, the powers of the alimentary canal improve, and the blood serum contains some antitoxic substance which is not present in patients in whom the disease is progressive, nor in the serum of healthy persons. Thus in seven out of ten patients the blood serum had a definite agglutinative action on bacillus coli. Nature, then, appears to check the disease and produce remissions by producing a hyperleucocytosis, and the leucocytes in their turn produce some substance of the nature of an antitoxin. Further, an attempt to produce remission was made as follows:—A patient in the second stage, during a well-marked remission, had an attack of lumbago. He was wet cupped, and the blood defibrinated. This was injected subcutaneously to the amount of two cb. cm. daily for three weeks, into two patients in an early and progressive stage of the disease. This was done two years ago. The first patient so far recovered that he was discharged, and supports himself at his trade, though still having some motor symptoms. The second patient showed physical improvement, but no mental change. The progress of his disease has, however, been definitely arrested for nearly two years. As it was obviously impossible to continue treatment on these lines, the author is now trying injections of blood serum of a horse immunised to bacillus coli. He concludes that general paralysis is directly due to poisoning by the toxins of bacteria, whose point of attack is through the gastric and intestinal mucous membrane. The poisoning is probably a mixed one, but the bacillus coli is apparently one of the noxious organisms, and the above recorded results suggest that some form of serum treatment is the proper one for this as yet incurable disease.

**Meningitis.**—Foulerton,[3] in the course of a paper on pneumococcic infection, says that all cases of epidemic meningitis may be put down as caused by the diplococcus intracellularis meningitidis, whereas sporadic cases may be due either to the above-named organism, or to the micrococcus pneumoniæ or to the streptococcus pyogenes—this is, of course, excluding syphilitic and tuberculous cases. Each of these three cocci may be the cause of the purulent form, whereas the sero-fibrinous forms of meningitis are due either to the micrococcus pneumoniæ or to the diplococcus intracellularis. The symptoms and gross morbid changes in these two forms of meningitis may be practically indistinguishable, and the differential diagnosis only possible by isolation of the causative parasite. With regard to the purulent forms of sporadic meningitis caused by any one of these three cocci, the position is very much the same, the symptoms and gross anatomical changes are identical whatever the causative parasite.

**Sporadic Cerebro-Spinal Meningitis.**—Hunter and Nuthall[4] have examined 10 cases of sporadic cerebro-spinal meningitis bacteriologically. They found in all cases a diplococcus in the cerebro-spinal fluid, and in nine cases also during life by lumbar puncture. This diplococcus had the same morphological and biological characteristics as Weichselbaum's diplococcus intracellularis meningitidis. In some cases the diplococcus was present in pure culture, in others associated with other micro-organisms—*e.g.*, bacillus influenzæ and bacillus tuberculosis. The clinical picture and pathological changes found in the cases are those met with in so-called "posterior basal meningitis," which is in all probability a sporadic manifestation of cerebro-spinal meningitis and produced by the same micro-organism. Two more or less definite varieties of the micro-organism were found, one of which grows luxuriantly on agar, glycerine agar and blood-serum, whilst the other presents a very delicate growth.

**Cerebro-spinal Meningitis.**—Buchanan[5] has recorded three unusual cases of cerebro-spinal meningitis which occurred in the course of an outbreak of the disease in an Indian prison. In the first, a man aged 40 was admitted to hospital complaining of pains in the joints. Both knees, both ankles, and the right wrist were swollen and painful. The temperature was 102°. The patient gradually became unconscious, with retention of urine and involuntary passage of fæces. He died on the sixth day. At the autopsy, the joints were full of flaky yellow fluid, and there was a purulent exudation over the brain. Such an arthritis is rare in cerebro-spinal meningitis, and it is quite exceptional for it to be the first recognisable feature. In the second case, a man aged 55 was admitted with a temperature of 102°, motor aphasia and right hemiplegia. During the six days of his illness the temperature varied from 101° to 102°, and on the second day before death herpes appeared on the right side of the upper lip. At the necropsy a thick yellow fibrinous exudation was found over both hemispheres. In the third case, a man aged 45 had worked all day and took his food at 6 P.M. Shortly after he vomited and quickly became unconscious, breathing became stertorous and the pupils dilated. An hour subsequently Kernig's sign was well marked. The patient died in three and a half hours. At the necropsy the surface of the brain was intensely congested, and the brain tissue seemed infiltrated with blood-clot. The fluid in the spinal canal looked almost like pure blood. Cultures showed the presence of the diplococcus intracellularis. This fulminant hæmorrhagic type of cerebro-spinal meningitis is very rare.

**Syphilitic Basal Meningitis.**—Hoffmann[6] has described a case of precocious syphilitic basal meningitis. The symptoms developed as early as 4½ months after the primary affection, and 2½ months after the secondary rash began; they consisted of, at first, pains in the right temple and occipital region; two days later he vomited, and was seized with short attacks of giddiness. Three weeks later it was found that the right facial nerve was markedly paralysed, and the right abducens slightly so. The taste sense was less acute on the right than the left side of the tongue, and the tongue was protruded to the right. There was also double optic neuritis. The symptoms were too localised for any intracerebral lesion, and were of too short a duration for any gummatous affection. The affection was therefore diagnosed as a circumscribed syphilitic basal meningitis. All the symptoms cleared up under anti-syphilitic treatment, except a slight paresis of the face.

[1] Brit. Med. Jour., June 29, 1901. [2] Brit. Med. Jour., June 29, 1901. [3] Brit. Med. Jour., Sept. 21, 1901. [4] Lancet, June 1, 1901. [5] Lancet, July 13, 1901. [6] Berl. Klin. Woch., March 18, 1901.

